# The Wall Apposition Evaluation for a Mechanical Embolus Retrieval Device

**DOI:** 10.1155/2018/9592513

**Published:** 2018-09-25

**Authors:** Xuelian Gu, Yongxiang Qi, Arthur G. Erdman

**Affiliations:** ^1^Shanghai Institute for Minimally Invasive Therapy, University of Shanghai for Science and Technology, 516 Jungong Road, Shanghai 200093, China; ^2^MicroPort Endovascular (Shanghai) Co., Ltd., 3399 Kangxin Rd., Shanghai 201318, China; ^3^Department of Mechanical Engineering, University of Minnesota, 111 Church St. SE, Minneapolis, MN 55455, USA

## Abstract

A computational evaluation approach to the wall apposition of a cerebral mechanical emboli retrieval device (MERD) is presented. The typical enclosed multilattice structure, manufactured from the thin-walled Nitinol tube, consists of repeated “V”-shaped unit cells. During interventional thrombectomy, the MERD system is delivered inside an artery stenosis segment to capture emboli and restore cerebral blood flow. The wall apposition, which deteriorates during embolus capture, occurs during system migration along the tortuous intracranial vessel. The commercial finite element analysis (FEA) solver ABAQUS 6.10 Standard and user subroutine (UMAT/Nitinol) are utilized to study the ability to remain in close contact with the curved vessel wall during migration. In this numerical analysis, the influence of the contacting interference loadings on structure deformation and strain field distribution is obtained and analyzed. The results indicate that the middle segment of the MERD seriously contracts or collapses inside the curved vessel. In addition, the peak strain is in the apex flow-prone region and maintains at the safe range.

## 1. Introduction

Stroke is a type of brain function disease which is due to the disturbance of blood supply to the brain tissue. Stroke has already become the second leading cause of death in the world, and approximately 88% is acute ischemic stroke (AIS) [1]. It is reported that the number of deep venous thrombosis cases has increased to 250,000 per year in the United States, and the ratio of morbidity or mortality is also fairly high [2]. During the past decade, mechanical embolus retrieval devices (MERDs) have been extensively used as an interventional neurovascular device to treat AIS [3–6]. The MERD is designed to capture the embolus or thrombus, scaffold the afflicted artery, and restore blood flow. This paper reports the analysis of the MERD which is fabricated from the nickel-titanium alloy thin-walled tube by laser cutting. After electropolishing and heat treatment for the shape setting, the embolus capture device is shaped like an enclosed tubular multilattice structural “funnel.” MERD structures vary in complexity from a 3D helical coil to a laser-cut nickel-titanium (Nitinol) tube. So far, several embolus retrieval devices have been used in clinics, such as the Merci Retrieval system (Concentric Medical, Mountain View, California, USA), the Penumbra Aspiration system and the Stent Retrievers, and others [5, 6]. The latest generation of mechanical thrombectomy devices is the ‘stent retriever' family: Solitaire (ev3 Endovascular, Plymouth, Minnesota, USA), Trevo Pro (Stryker Neurovascular, Kalamazoo Michigan, USA), and arguably the Penumbra 3D separator (Penumbra, Alameda, California, USA) [7–9].

**Figure 1:**
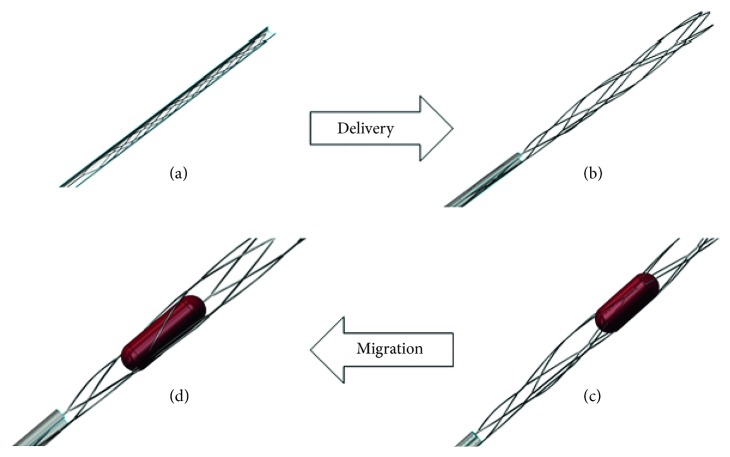
The numerical workflow of the MERD. (a) Crimp. (b) Deploy. (c) Extract. (d) Capture.

In clinical application, the MERD works according to the following steps:

1. Crimp the stent into a catheter and delivery to the stenosed (blocked) vessel, as shown in Figure 1
2. Deploy to capture the embolus while the outer catheter is removed, as shown in Figures 1 and 1
3. Migrate the embolus removal device and extract tissue from the patient's body, as shown in Figure 1.

The cerebral blood vessels have a relatively small profile and often small radius of curvature in the anatomy. After the MERD has been inserted and has migrated inside the tortuous cerebral vessel, the embolus retrieval performance is of great importance [3, 5]. The wall apposition, which describes the stent or MEDR's ability to remain in close contact with the adjacent vessel wall [3], is a significant mechanical requirement of a MERD system while it is deployed in a curved vessel and will affect the effectiveness and accuracy of embolus retrieval.

So far, few researches have been published to study the mechanical behavior of a thrombectomy device with a numerical modeling method. However, the mechanical performance of multilattice tubular devices, such as vascular stents, has been studied already [3, 10–15]. Krischek et al. proposed the mechanical features for a MERD, including the radial force, wall apposition, conformability, and Gator backing (Gator backing describes a stent's tendency to flair its struts outward, forming protrusions, when the stent is placed around a bend) and carried out experimental tests [3]. Kleinstreuer et al. simulated crimping, deployment, and cyclic-loading procedures. A high-cycle fatigue prediction method for mean strain/alternating strain in Nitinol material was established in [10]. Kate et al. compared nonstent retriever and stent retriever mechanical thrombectomy devices for the endovascular treatment of acute ischemic stroke. Stent retriever mechanical thrombectomy devices achieve higher recanalization rates than nonstent retriever devices in acute ischemic stroke with improved clinical and radiographic outcomes and safety [11]. Azaouzi et al. analyzed the deployment of a self-expanding stent inside an artery by FEA, and the results could be used to assess the impact of the stent on the artery and the influence of the artery on the deformation field within the stent [12]. Gu et al. presented a numerical analysis of a semienclosed tubular mechanical embolus retrieval device (MERD) for the treatment of AIS, and the FEA methodology is used to evaluate mechanical performance and provide suggestions for optimizing the geometric design [13]. Wu et al. generated a finite element (FE) model of a vascular stent with tetra-elements. The axial flexibility of the stent is studied by applying moment in [14]. Grogan et al. designed CoCr stents and compared the mechanical properties of the deployment, radial force, longitudinal resistance, and flexibility. The difference between a rigid expanding tool and a polymer balloon is demonstrated in [15].

After being delivered to the targeted artery wall, Nitinol stents expand and contact with the artery wall. The tubular stent is designed to be embedded in the stenosed blood vessel segment to avoid movement. The multilattice MERD, however, is manipulated so that it migrates inside the blood lumen to capture and retrieve the plaque blockage, as shown in Figures 1 and 1. Poor wall apposition inside tortuous vessels may result in undesired clinical accidents and even failure to retrieve plaque. So far, the numerical simulation process of embolus retrieval in tortuous vessel walls has not been reported. In interventional thrombectomy, the Nitinol MERD functions as a disposable surgical instrument rather than a permanent implant. Prediction of structural failure depends on the peak strain value. Finally, the unique design of the distal and proximal structure constructed is demonstrated. The paper develops a scientific numerical analysis workflow of the MERD for the expansion capability and wall apposition. The analysis method provides a structural optimization scheme for MERD design. In clinical use, an operational guideline for embolus retrieval and migration of the MERD is demonstrated, which will help optimize a specific procedure. In conclusion, the study of the MERD for the wall apposition inside a tortuous blood vessel is provided to improve the device's mechanical capability as well as to aid in surgical training.

In this paper, a typical tubular enclosed mesh-like MERD model is built to study the influence of shape setting and migration. ABAQUS 6.10/Standard (DS SIMULIA, RI, USA) commercial FE code and its user material subroutine (UMAT/Nitinol) are employed to simulate the procedure and the MERD/artery contact interaction mechanism.

Our study presents the following critical measurement of performance: (1) large strain distribution and the highest peak value of maximum principal strain (MPS) in the microstructure and (2) wall apposition performance along the tortuous artery in the macrostructure.

The analysis results are used to assess the safety and efficiency of the MERD. The wall apposition performance evaluation methodology is a scientific numerical method to analyze preexisting structural flaws and offer approaches for design optimization.

### 1.1. Methods

**Figure 2:**
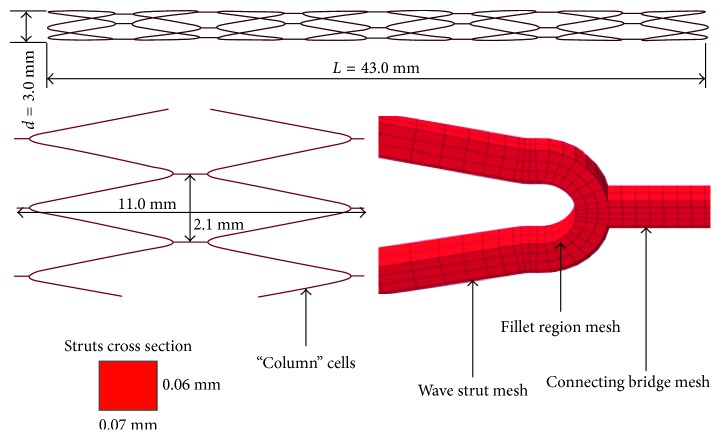
Geometry parameters and mesh model of the MERD.

A 3D finite element model has been generated to study the effects of MERD expansion and migration. The numerical simulation processes are achieved with the contact interactions of a MERD/shape-setting cylinder, MERD/expanding cylinder, and MERD/artery at a body temperature of 37°C [10]. The geometry and mesh model of the embolus removing device are shown in Figure 2.

**Figure 3:**
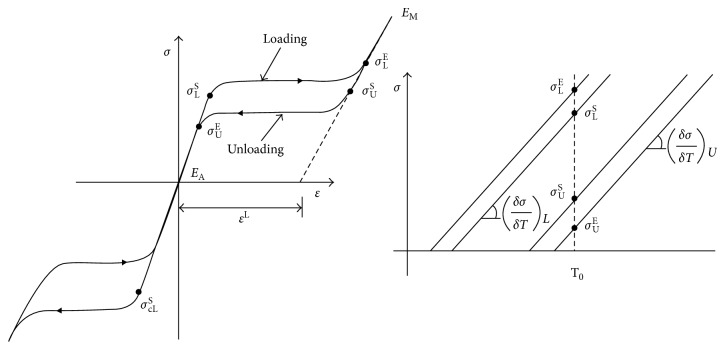
Nitinol material properties (from ABAQUS Nitinol UMAT) [10].

In the numerical analysis, the MERD is assumed to be a homogeneous isotropic incompressible deformed body in the absence of residual stress. The shape-setting cylinder and expanding cylinder are designed to be a semirigid movable cylinder shell. The tortuous cerebral vessel is modeled as a “C”-shaped discrete rigid shell. The Nitinol material constitutive model is characterized by the ABAQUS 6.10 software (UMAT/Nitinol) user subroutine.  Figure 3 provides the stress-strain and stress-temperature curves for the template of Nitinol alloy based on ABAQUS software, while Table 1 lists the specified parameters of Nitinol material from the previous research [10]. These parameters demonstrate the distinct mechanical behavior of Nitinol during loading and unloading conditions with specific temperature.

**Figure 4:**
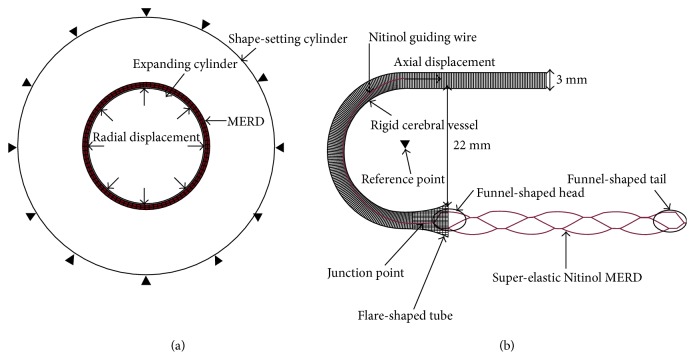
Schematic points of assembly boundary conditions. (a) Expansion for shape setting. (b) Pulling and migrating inside the tortuous cerebral vessel.

This mesh-like tubular MERD comprises five “column” cells in the axial direction. Each “column” cell consists of four “V”-like wave rings, and each “column” cell is connected with connected bridges circumferentially. The strut thickness, width, original outer diameter, nominal final outer diameter, artery centerline radius, and artery cross-profile diameter are 0.07, 0.07, 2.00, 4.00, 5.00, and 3.00 mm, respectively. The element types C3D8I (three-dimensional eight-node Stress Hex incompatible element), SFM3D4 (three-dimensional four-node quadrilateral surface element), and R3D4 (three-dimensional linear four-node bilinear rigid quadrilateral element) are utilized for the MERD, tortuous vessel, and expanding/shape-setting cylinder, separately. To minimize the influences of mesh density, two layer elements for the wall thickness [16] and 16 elements along the fillet edge are used. The element and node numbers of the MERD FE model are 40,000 and 20,000, respectively. In addition, to form an enclosed thrombus-capturing “cage,” “funnel”-shaped tails are meshed and connected to the MERD's FE principal body after expansion and annealing. At the end of shape setting, a Nitinol tortuous guiding wire is tied to the “funnel”-shaped head for the pulling/pushing simulation. A flare-shaped tube is connected to the “C”-shaped cerebral vessel. The novel structural design offers smooth and stable surface interaction to avoid computational contact divergence. The boundary conditions for shape setting and migration are described in Figure 4.

In the shape-setting step, a radial outward displacement is imposed on the expanding cylinder to achieve the expanded shape size. Besides, it is also constrained axially and circumferentially, that is, preventing the transitional and rotational movements. Meanwhile, the shape-setting cylinder is restrained in all DOF to avoid offset. A single node of MERD instance is fixed axially to restrict movement. In the migration step, the reference point of a rigid artery is restrained in all DOFs under a global rectangular coordinate system. Negative axial displacement, imposed on the guiding-wire side, is used to pull the MERD along the desired tortuous artery.

The master/slave contact pair algorithm is utilized to build the contact interface between the expanding/shape-setting cylinder and the MERD. Meanwhile, the arterial inner surface is taken as the master surface, and the MERD outer surface is set as the slave surface. The penalty contact method is employed. It is notable that self-contact interaction should also be considered to avoid struts overlapping during the migration step. In addition, a damping factor could be employed discreetly to stabilize the contact-induced vibration behavior, improve convergence, and reduce computational expense in contacting interaction domains.

## 2. Results

### 2.1. Validation

**Figure 5:**
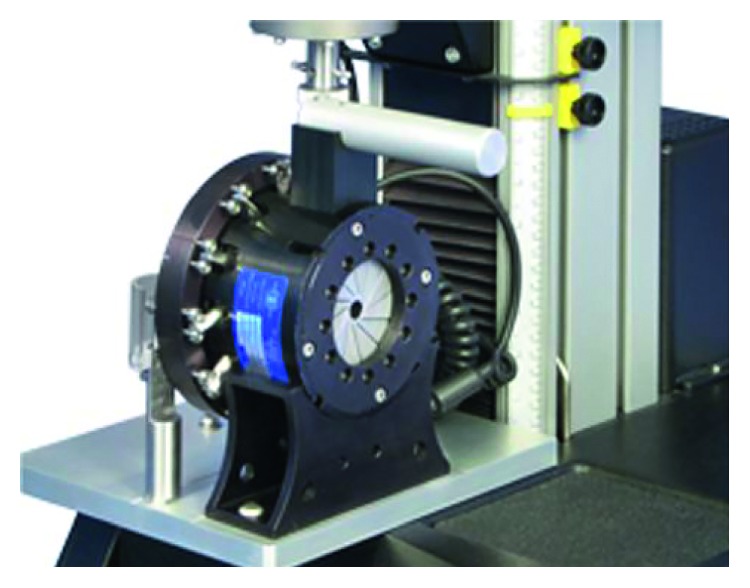
The electrical force sensor (RX500, Machine Solutions Inc.).

**Figure 6:**
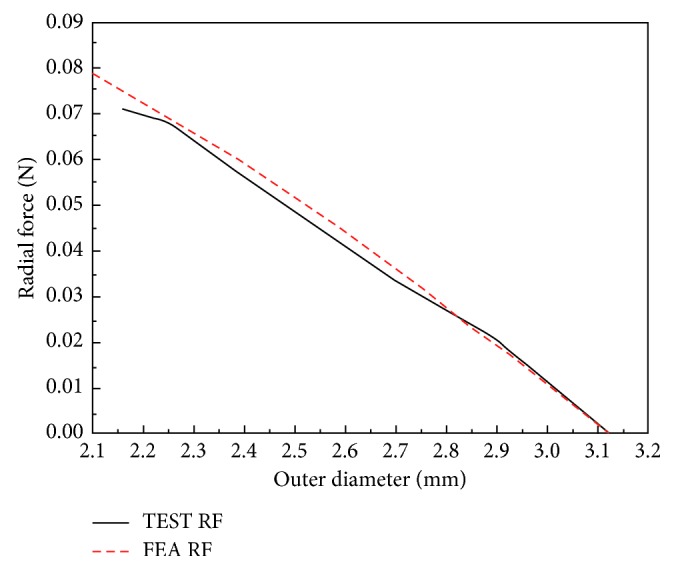
Experimental validation of FEA.

To validate the finite element model and computational algorithm, a diamond-shaped pattern is generated for the numerical simulation and experimental test. The comparison between test and FEA radial forces is used to predict the accuracy and reliability. In the experimental test, the measurement system applies displacement on several metal slices to compress the multilattice pattern. The electrical force sensor (RX500, Machine Solutions Inc.), shown in Figure 5, obtains and reflects the real-time support force value. For the radial force (RF) curve plots, the outer diameter of the MERD is designed as the horizontal *x*-axis, while the vertical axis prescribes the balanced force value. In the progress of numerical simulation, the crimping slice is replaced by a rigid removable cylinder to compress the original pattern. The curves for the test and numerical process are shown in Figure 6.

The result indicates that the test and simulation curve match well; that is, all of the numerical outcomes are relatively accurate and could therefore provide a reliable performance analysis and structure optimization report.

### 2.2. Shape Setting

**Figure 7:**
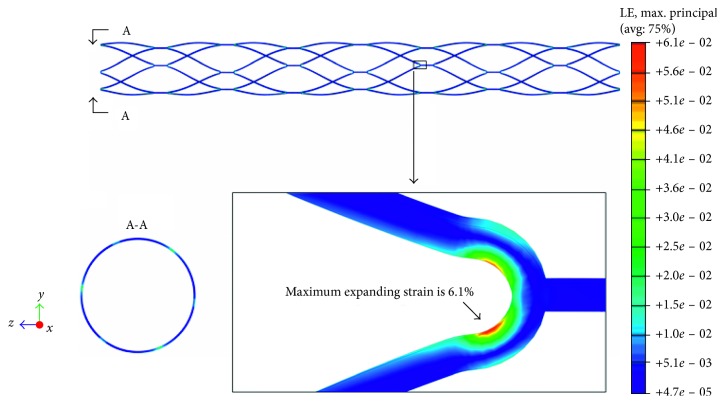
Maximum principal strain contour plots of MERD expansion: global and detailed schematic views.

The original laser-cut tubular multilattice structural MERD is expanded by imposing an outward radial displacement on a cylinder surface. Once the final shape is accomplished, the mechanical behavior of cross-profile expansion and axial length shrinkage is presented in Figure 7.

During shape setting, the obtained radial outer diameter ranges from 2.00 to 4.00 mm, and the axial length ranges from 42.9 to 40.0 mm. In contrast to traditional metallic material, the Nitinol material fracture and fatigue failure are strain induced [10]. Structural damage can occur from outright fracture during the expansion step. Figure 7 presents the strain field contour plots of the expanded MERD. It indicates that high-magnitude strains are always located in the strut's inner fillet tensile side and its vicinity. The strain distributions across the MERD middle segments are negligible. The peak strain located in the “flow-prone” region achieves a value of 6.1%, which is much lower than the critical strain threshold for Nitinol of 12% [10]. Therefore, no issues of cracks or fractures in the structure are likely in the shape-setting step.

### 2.3. Migrating

**Figure 8:**
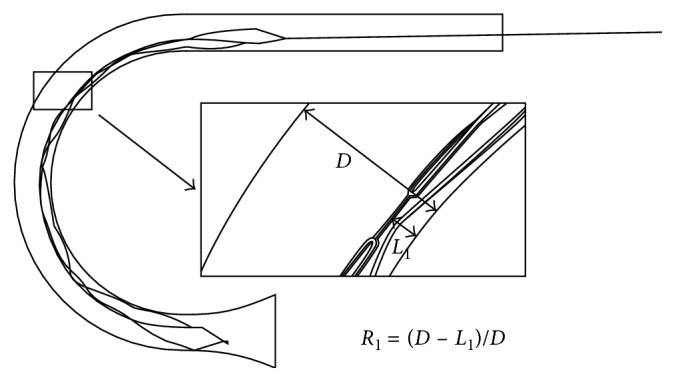
Wall apposition performance of a MERD segment in parametrical schematic.

MERD struts may seriously contract or collapse due to contact-dominant bending in the tortuous blood vessel. The wall apposition performance is a significant criterion to evaluate the device effectiveness during migration and embolus capture.  Figure 8 illustrates a significant cross-profile contraction of the middle segment struts while they are being pulled along a curved path.

**Figure 9:**
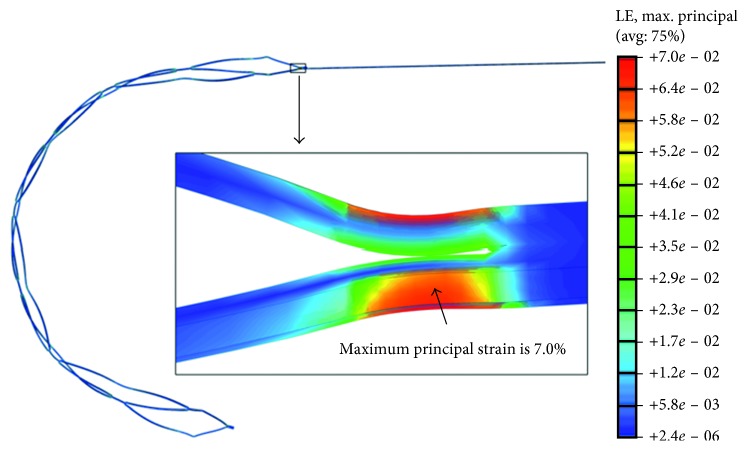
MPS field contour plots of MERD migration along a tortuous artery: global and detailed schematic views.

The formula for the cross-profile reduction ratio is calculated and proposed as below:

(1) $$\begin{array}{c} R_{1}=\frac{\left(D-L_{1}\right)}{D}, \end{array}$$

where *D* is the artery cross-profile diameter, *L*_1_ is the radial contraction lengths, and *R*_1_ is the cross-profile reduction ratios. As a result, *D* obtains a value of 3.0 mm, *L*_1_ 0.8 mm, and *R*_1_ 73.3%. The strain field distribution contour plots of the deformed MERD are displayed in Figure 9.

Figure 9 indicates that high-magnitude MPS is always located in the shoulder of the constrained strut's apex. In the migration step, a structure-induced resultant force is assumed to be applied to stretch and straighten the apex struts. The peak strain located on the ‘funnel'-shaped head reaches a value of 7.0%. Compared to a Nitinol ultimate tensile strength (UTS) critical threshold of 12% [10], there is no risk of crack or fracture failure during the migration step. All other areas appear to have relatively low strain. They are also confirmed to stay in the safe domain of Nitinol alloy. It has been speculated that the former highest peak MPS appears on the basis of unsmooth geometry [17] and a tiny connecting section in the microstructure.

## 3. Discussion

This paper explores the biomechanical concept of the wall apposition performance for tubular MERD migration simulation. This behavior is regarded as reflecting the safety of pulling migration as well as the effects of embolus removal. Structure designers can simulate and analyze the virtual prototypes. Various topology and dimension optimization methods could also be used to provide closer contact with the tortuous vessel. As a vital mechanical index, the numerical wall apposition performance could offer surgeons a guideline for a reasonable MERD choice.

The contraction or collapse behavior is predicted to be induced by the bend resistance deficiency of the tubular axial structure. The key factors of the desired mechanical performance include the strut width, strut thickness, wave axial length, unit cell number, and connecting-bridge topology. The wall apposition numerical analysis of the MERD has been displayed in the absence of an arterial constitutive model and anatomical geometry. The simulation process of thrombus capture is also not presented. The simulation results can be applied to provide a reference for the design of stent retriever mechanical thrombectomy devices which achieve higher recanalization rates than nonstent retriever devices in acute ischemic stroke with improved clinical and radiographic outcomes and safety [11].

Based on the analysis above, a number of recommendations are suggested for engineers for optimization of MERD structure. For example, a shorter independent support unit cell could undergo and resist higher contact-induced axial bending loading. Therefore, an approach of axial length shortening can be utilized to improve the structure performance and minimize cross-sectional collapse effects. The geometry of the former highest peak MPS region should be optimized. To reduce the strut's apex strain, the strut's external side (the so-called “shoulder” region) and internal side (close to the fillet curve region) should be smooth and continuous. The radius of curvature of the fillet region should also be adjusted. Above analyses will provide valuable information about the MERD design and struts dimensions that can be optimized in order to maximize the effectiveness of the MERD during the shape-setting and migrating process.

In future work, a particular cerebral vessel wall will be three-dimensionally reconstructed and modeled on the basis of patients' MRI images. The cerebral vessel wall and emboli will be modeled as homogeneous isotropic hyperelastic materials. Two types of MERDs will be generated and simulated for the migration and embolus removal in the reconstructed cerebral vessel. During interventional mechanical thrombectomy, the management of the delivered system deployment and embolus capture is considered crucial and difficult to optimize. The method provided in the paper is suggested as an operational guideline for modeling accurate MERD insertion during clinical use. Based on the demonstrated axial shrinkage during MERD self-expansion, physicians can deliver more confidently the embolus device system to the correct position in a diseased cerebral vessel. In this way, the MERD will self-expand and capture plaque emboli more reliably. It will be better if some experiments are adopted to verify the results in the future.

## 4. Conclusion

Our study suggests that the independent support unit cell should be reduced to resist higher contact-induced axial bending loading. An approach of axial length shortening can be utilized to improve the structure performance and minimize cross-sectional collapse effects. The geometry of the former highest peak MPS region should be optimized. In addition, these findings favor the use of verifying the performance of the delivery system insertion and the effectiveness of embolus retrieval [13].

## Figures and Tables

**Table 1:** Material parameters of Nitinol.

Parameters	Values
Austenite elasticity, *E*_A_ (MPa)	51700
Austenite Poisson's ratio, *ν*_A_	0.3
Martensite elasticity, *E*_M_ (MPa)	47800
Martensite Poisson's ratio, *ν*_M_	0.3
Transformation strain, *ε*^L^	0.063
Start of transformation loading, *σ*_L_^S^ (MPa)	600
End of transformation loading, *σ*_L_^E^ (MPa)	670
